# Usability, Acceptability, and Usefulness of an mHealth App for Diagnosing and Monitoring Patients With Breakthrough Cancer Pain

**DOI:** 10.2196/10187

**Published:** 2019-04-01

**Authors:** Jaime Boceta, Daniel Samper, Alejandro de la Torre, Rainel Sánchez-de la Rosa, Gloria González

**Affiliations:** 1 Unidad de Hospitalización Domiciliaria y Cuidados Paliativos Servicio de Medicina Interna Hospital Universitario Virgen de la Macarena Sevilla Spain; 2 Servicio de Anestesiología Clínica del Dolor Hospital Germans Trias i Pujol Badalona Spain; 3 Servicio de Oncología Radioterápica Hospital Universitario Puerta del Hierro Madrid Spain; 4 Medical Department Teva Pharmaceutical Madrid Spain; 5 Adelphi Spain Barcelona Spain

**Keywords:** breakthrough cancer pain, mHealth, mobile app, App INES·DIO

## Abstract

**Background:**

Breakthrough pain is a major problem and a source of distress in patients with cancer. We hypothesized that health care professionals may benefit from a real-time mobile app to assist in the diagnosis and monitoring of breakthrough cancer pain (BTcP).

**Objective:**

This study aimed to test the usability, acceptability, and usefulness in real-world practice of the mobile App INES·DIO developed for the management of patients with BTcP.

**Methods:**

This study consisted of a survey of a multidisciplinary sample of 175 physicians who evaluated the mobile app after testing it with 4 patients with BTcP each (for a total of 700 patients). The digital profile of the physicians, use of the different resources contained in the app, usefulness of the resources, acceptability, usability, potential improvements, intention to use, and additional resources to add were recorded.

**Results:**

Of the 175 physicians, 96% (168/175) were working in public hospitals. They had an average of 12 (SD 7) years of experience in BTcP and almost all (174/175, 99.43%) had an active digital profile. The Eastern Cooperative Oncology Group and Karnofsky performance scales, the Visual Analogue Scale, and the Davies algorithm to diagnose BTcP were the most frequently used tools with patients and were assessed as very useful by more than 80% (140/175) of physicians. The majority (157/175, 90%) answered that App INES·DIO was well designed and 94% (165/175) would probably or very probably recommend it to other colleagues. More than two-thirds indicated that the report provided by the app was worth being included in patients’ clinical records. The most valued resource in the app was the recording of the number, duration, and intensity of pain flares each day and baseline pain control to enhance diagnosis of BTcP. Additional patient-oriented cancer pain educational content was suggested for inclusion in future versions of App INES·DIO.

**Conclusions:**

Our study showed that App INES·DIO is easy to use and useful for physicians to help diagnose and monitor breakthrough pain in patients with cancer. Participants suggested the implementation of additional educational content about breakthrough pain. They agreed on the importance of adding new clinical guidelines/protocols for the management of BTcP, improving their communication skills with patients, and introducing an evidence-based video platform that gathers new educational material on BTcP.

## Introduction

### Background

Pain is one of the most prevalent health-related concerns and most common clinical conditions for seeking medical help [1]. In cancer patients, pain is a frequent and distressing symptom, which occurs in up to 40% of patients in the early stages of the disease and rises to 70%-90% in its most advanced stages [2-4]. Despite adequately controlled background pain, many patients experience transient exacerbations of severe pain, known as breakthrough cancer pain (BTcP), a complicated state of pain that negatively impacts patients’ quality of life and provokes intense suffering. Indeed, BTcP episodes are associated with increased levels of depression and emotional disorders, interfering with other aspects of the disease, and result in higher health care costs both for patients and society [5,6].

With the aim of improving BTcP management, the Spanish Society of Medical Oncology published recommendations in 2013 for the diagnosis and treatment of BTcP and launched a program for the diffusion and implementation of these recommendations [7]. However, even today there is no unanimous consensus among specialists on the clinical features for defining BTcP.

Factors considered in the definition of BTcP as well as the procedures for its diagnosis, assessment, and monitoring may influence the choice of a treatment and consequently, patient outcomes. Hence, it was important to obtain a consensus on these issues from a broad group of experts in cancer pain.

Recently, Boceta et al published the results of a two-round Spanish multicenter exploratory Delphi study that investigated the opinion of an expert panel in cancer pain to conclude how to define, diagnose, assess, treat, and monitor BTcP [8]. The study intended to seek consensus in the definition of BTcP and identify the challenges regarding a set of recommendations for the complete management of BTcP in clinical practice. Regarding the clinical aspects for diagnosing BTcP, it was generally agreed that (1) background pain should be controlled, but not necessarily with opioids, (2) there must be exacerbations (no matter whether the number of flares per day are ≥4 or not), (3) the duration of an episode should be ≤1 hour, (4) intensity of pain greater than 7 out of 10, and (5) it is not considered the same as an end-of-dose effect. The Davies algorithm was recommended for diagnosing BTcP. All these recommendations should be followed in the day-to-day clinical practice to enhance the management and control of patients with BTcP.

The results of the Delphi study were used for the development of a real-time mHealth cancer pain app named App INES·DIO (the abbreviation in Spanish for Instrument for the Assessment and Monitoring of Breakthrough Cancer Pain).

Internet-based and mHealth apps are transforming how people monitor, manage, and communicate health-related information [9]. mHealth supports public health interests through the use of mobile devices [10,11]. Mobile apps to improve health are proliferating, but before health care providers or health care organizations can recommend an app, strategies for evaluating them are necessary. More primary research is needed to identify apps that are effective, provide accurate information, and are user-friendly [12].

### The App INES·DIO

App INES·DIO was developed by an international information technology expert company (Virtualware, Bizkaia, Spain), which was licensed by the Spanish Agency of Medicines and Medical Devices in 2014. The contents of App INES·DIO as well as the test phase of the app were the responsibility of Adelphi Spain, a health and marketing research group. Of note, the usability testing will be removed from the mHealth app as this study phase is completed, and the name of the app, when commercially launched, will be different.

With the rise of smartphone usage in the medical field, the Food and Drug Agency announced in 2013 that it would regulate mobile medical apps to protect users. European and other regulatory agencies soon followed suit [13]. App INES·DIO is certified as a CE-mark Class-1 medical device used to produce or change data on individual cancer patients with the aim of a better management and control of the breakthrough pain.

It has been reported that native mobile apps are better accepted by end users than webpages or Web apps and provide better support for customization of device characteristics [14,15]. Our idea was to create a mobile app able to run as a native app on various mobile platforms and operating systems (eg, Android, iOS). The content of App INES·DIO gathered the most significant results of a Spanish Delphi study about the consensus and controversies in the definition, assessment, treatment, and monitoring of BTcP [8]. This app allows the physician to generate an individual patient register to be included (via email) in the patient’s clinical history.

**Figure 1:**
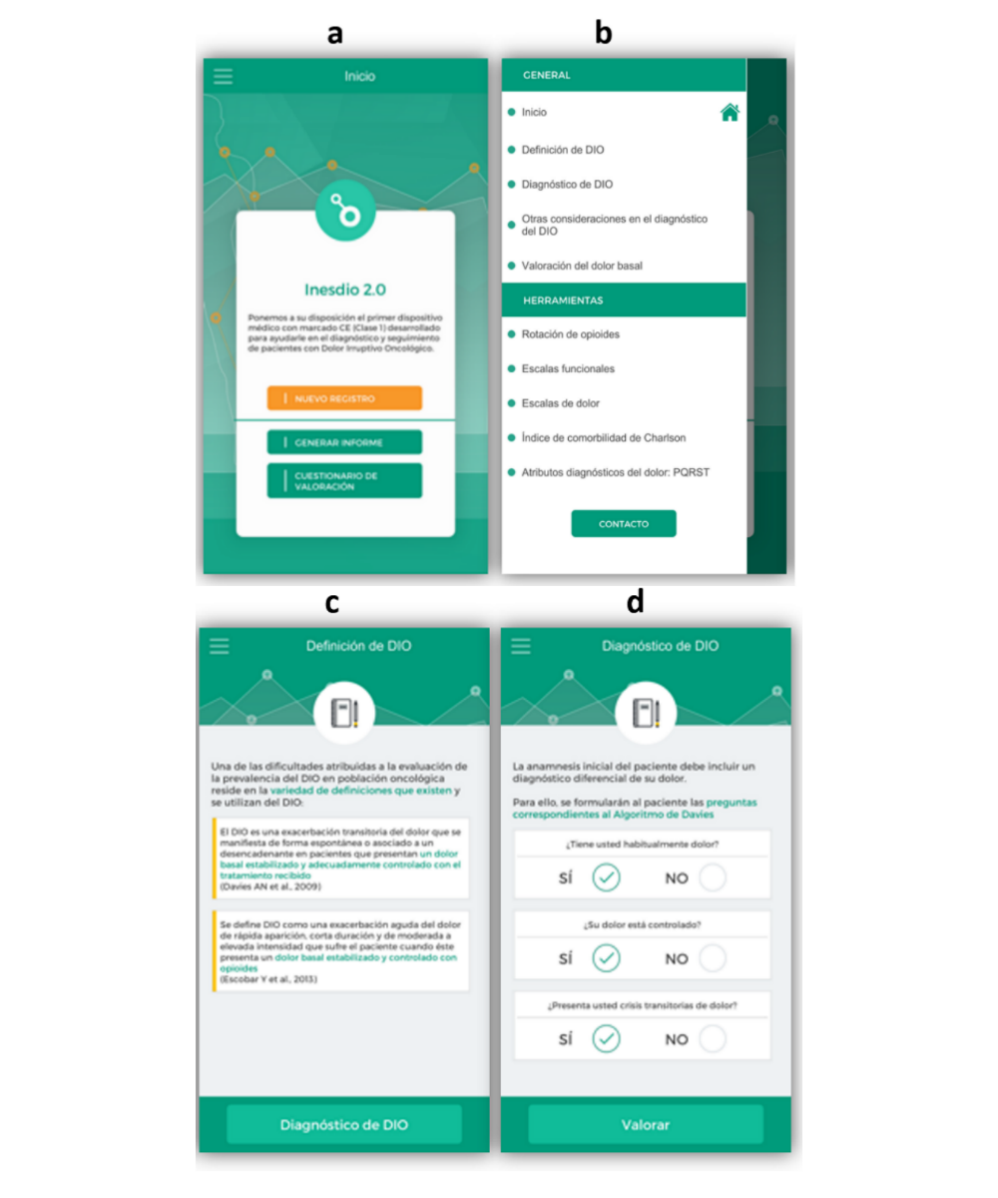
Screenshots of App INES·DIO: a) starting workflow of the app, b) general information and toolbar for a new patient registry, c) definitions of breakthrough cancer pain (BTcP), d) diagnosis of BTcP.

**Figure 2:**
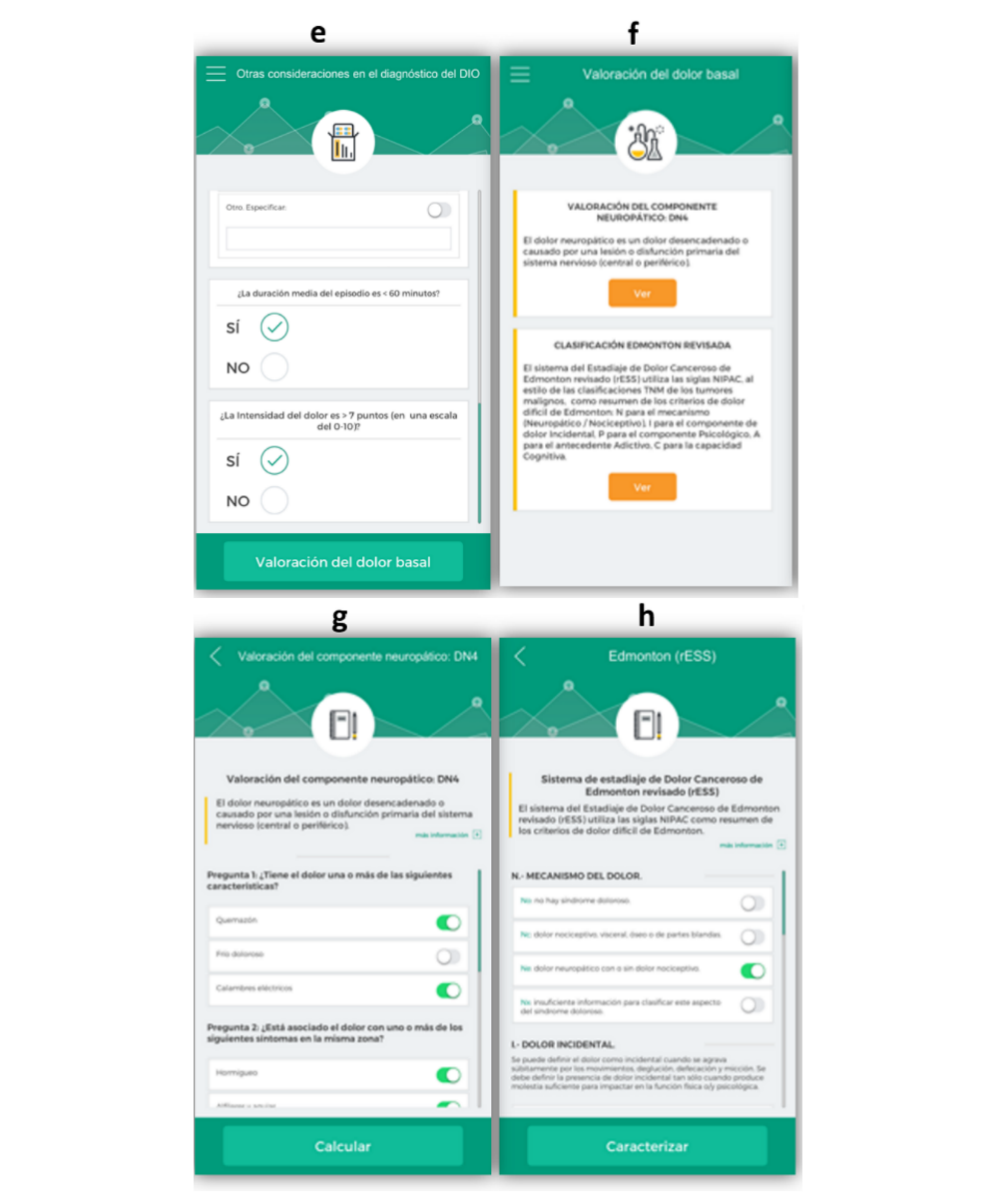
Screenshots of App INES·DIO: e) other considerations for diagnosing breakthrough cancer pain (BTcP), f) evaluation of baseline pain, g) diagnosing neuropathic pain, and h) Edmonton’s Classification of cancer pain.

The app development process was conducted following three steps: (1) enter a new case (ie, use the app with a new patient) with complete information on the breakthrough pain, (2) create a new report with all input data on the cancer patient, and (3) complete an assessment test related to the usability of the app (Figures 1 and 2). This last step will be no longer available upon the completion of this study and will therefore not be present in the future version of the App INES·DIO. The app was developed in the Spanish language.

**Figure 3:**
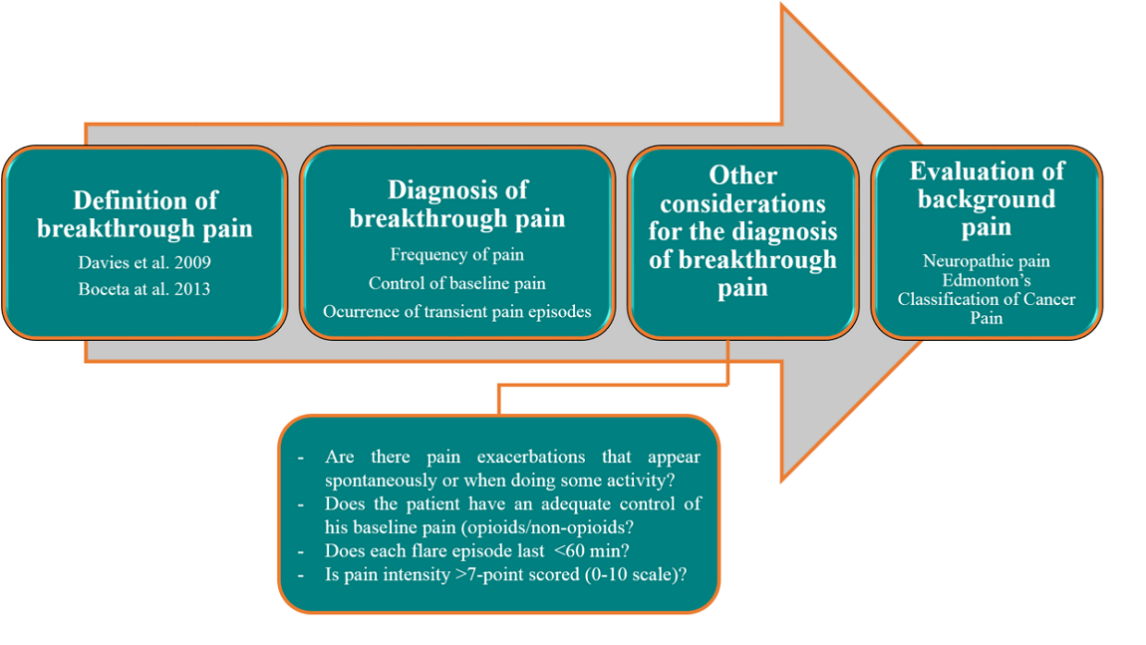
App INES·DIO workflow that guides a clinician to open a new patient registry.

When launching the app, the user is requested to open a new registry for each patient and to go through four sequential steps (Figures 1 and 2). The workflow of a new register is summarized in Figure 3. After reading two different definitions of BTcP (Figures 1 and 2), additional information related to the Davies algorithm for BTcP diagnosis (ie, frequency and control of baseline pain, and the occurrence of transient pain episodes) is introduced (Figures 1 and 2). Davies et al [6] defined BTcP as a transitory exacerbation of pain that occurs, either spontaneously or may be associated with predictable factors, even though the baseline pain is relatively stable and well controlled. In line with Davies’ definition, Escobar et al [16] adopted the term “breakthrough pain” to describe a sudden and transient exacerbation of pain of high intensity and short duration (<20-30 minutes), which appears over the baseline of a stable persistent pain, when this has been reduced to a tolerable level by the use of strong opioids [6,16,17]. Both definitions allow us to distinguish BTcP from end-dose pain flares and those flares that occur during the drug analgesics titration of the background pain.

The clinician is then asked to fill in other considerations for a better diagnosis of BTcP (Figures 1 and 2). Once this last item is completed, the app immediately allows remote clinicians to assess each patient’s baseline pain (Figures 1 and 2). They are then asked to evaluate the neuropathic component of cancer pain using the DN4 questionnaire (Douleur Neuropathique 4) (Figures 1 and 2) and the last revised Edmonton Classification System for Cancer Pain (Figures 1 and 2).

Additionally, below the general information compiled to diagnose BTcP when registering a new patient, there is a toolbar incorporated into the app to help physicians diagnose and monitor patients with BTcP (Figures 1 and 2). Tools included were as follows: (1) Opioid rotation refers to a switch from one opioid to another in an effort to improve the response to analgesic therapy or reduce adverse effects, (2) Functional scales to assess the quality of life of cancer patients: the Karnofsky index (an attempt to quantify cancer patients’ general well-being and activities of daily life) and the Eastern Cooperative Oncology Group (ECOG) Scale (standard criteria for measuring how the disease impacts cancer patients daily living abilities such as ability to care for themselves, daily activity, and physical ability like walking, working, etc), (3) Pain rating scales include the Visual Numeric Scale (VNS), which is a segmented numeric version of the Visual Analog Scale (VAS) in which a respondent selects a whole number (0=no pain, 10=worst pain) that best reflects the intensity of pain, and the Categorical Scale (CS) (none/mild/moderate/severe), used only when the patient is not able to self-assess pain with any of the former scales, (4) the Charlson Comorbidity Index, which predicts 10-year survival in patients with multiple comorbidities (ie, age, acute myocardial infarction, congestive heart failure, peripheral arterial disease, cerebral-vascular disease, dementia, chronic pulmonary disease, connective-tissue disease, peptic ulcer, liver disease, diabetes mellitus, hemiplegia, renal failure, solid tumors, blood malignancies, and AIDS), and (5) PQRST pain assessment questions (P: provocative and palliative factors; Q: qualitative description of pain, “What does it feel like?”; R: region and radiation of pain; S: severity or intensity of pain after being scored by means of VNS and CS; T: timing or pain changes over time).

It is both interesting and critical to evaluate and improve information and communication technology tools before trying to distribute them. The ergonomic approach consisting of evaluating first in order to improve later may fulfil this goal. We therefore used an ergonomic framework where the quality of the App INES·DIO was defined by its usability, acceptability, and usefulness. These three elements have been already defined elsewhere [18]. Usability refers to ease of use and can be evaluated with criteria such as efficiency, acceptability (to address each physician’s desire to use App INES·DIO in the future), and lastly, usefulness (ie, relevance or efficacy), answering the question of whether the app allows physicians to reach their goal in BTcP management. The primary objective of this study was to carry out first-of-its-kind testing on App INES·DIO to understand usability, acceptability, and usefulness in real-world practice as well as the need to include new information and recommendations for better care of cancer patients with BTcP.

## Methods

### Study Design

To evaluate App INES·DIO, we performed a survey research study of both the mobile phone and tablet computer versions of the app. This research consisted of testing the usability of a novel prototype to validate the acceptability and usefulness of mobile app tools in the daily clinical practice of patients with BTcP.

Usability testing was conducted using a structured questionnaire to collect responses to 33 questions divided into three different blocks: (1) demographic and professional profile of participants, including gender, age, professional background, experience in treating BTcP patients, (2) participants’ digital profile, focused on previous experiences with mobile phone and tablet devices, and previous use of mHealth apps, and (3) a patient-related questionnaire based on those clinical features that could help when diagnosing and monitoring BTcP in cancer patients. At the end of testing period, participants were asked to answer follow-up questions about the app design and its features, its overall usefulness, their intention to use it in other type of patients (not only those BTcP-related), the acceptability of the mobile app and its features in everyday health management, and new interesting content to be included in the mobile app in the future.

### Participants

Our study sample consisted of 175 medical doctors from all over Spain from different health care units: medical oncology (n=66), radiation oncology (n=48), palliative care (n=42), pain units (n=18), and others (n=1). Participants worked in public hospitals (96%) and were highly experienced in BTcP (>12 years with more than 412 patients attended in the last year).

Every participant was asked to test the usability and the value in the clinical setting of App INES·DIO in 4 cancer patients each (this makes a total of 700 patients), with a different clinical profile of BTcP: newly diagnosed or in follow-up.

### Data Analysis

A descriptive study of the variables was carried out according to their type. For numeric variables, measures of central tendency and dispersion (eg, sample size, mean, median, minimum, maximum, standard deviation, 95% confidence interval) were applied. For the categorical variables, frequency distribution tables and percentages (n, %) were provided.

To evaluate some of the answers, a 7-point Likert-type rating scale was used (1=strongly disagree/never/never recommend, and 7=strongly agree/ always/always recommend).

## Results

### Professional and Digital Profile of Participants

The survey showed 48.6% of participants (85/175) were female and 51.4% (90/175) male, and 37.6% of participants (64/175) were between 36-45 years old. Over three-quarters (134/175, 76.6%) of the sample were physician assistants, and all BTcP-related medical specialties were represented among participants. Panelists mostly worked at public health care centers (168/175, 96%) and half of them (87/175, 49.7%) in large hospitals (≥500 beds). Participants had >12 years (SD 7) experience managing patients with BTcP, with an average of >400 patients attended during the last year. About a fifth (36/175, 20.5%) of physicians recruited for the study had also participated in the previous Delphi consensus study [8]. Most of the sample (155/175, 88.9%) was aware of the recommendations and (150/175, 85.5%) considered them to be useful for their clinical daily practice. Table 1 shows the digital profile of the sample. All participants owned a private mobile phone and had access to different types of apps for private use (ie, maps, email, press news, instantaneous communication platforms) and professional use (20% of downloaded apps are for clinical use). The most commonly used function on the Web related to at least one social network (ie, Facebook, LinkedIn).

### Mobile App Intervention in Patients

App INES·DIO was tested by 175 panelist clinicians after using it with 700 patients (4 patients per participant). Patients had been diagnosed with BTcP on average 3.77 months before this study. The app was mainly used to help physicians during their visit with cancer patients (79/175, 45.1%), followed by the course of BTcP flares (48/175, 27.6%), diagnosis of BTcP (42/175, 24.3%), and drug titration/change of treatment to control BTcP (39/175, 22.3%).

As described above, when initiating the app, every clinician was requested to open a new profile for each patient, going through four sequential steps to collect relevant clinical information for an enhanced diagnosis procedure of BTcP. After testing the different levels of usage of this diagnostic workflow, the BTcP definition by Davies et al [6] and Escobar et al [16], along with the Davies algorithm were shown to be used most frequently (Table 2). For defining each patient’s baseline pain, the DN4 neuropathic scale and Edmonton scale were used by 69.5% (121/175) and 63.4% (111/175) of participants, respectively.

The panel also rated the usability of the tools incorporated into the app to help physicians monitor pain, functional performance, and comorbidity of BTcP patients (Table 2). Both pain-rating and functional assessment scales were the most frequent tools used by clinicians, with a peak of 93.3% (163/175) for the VNS followed by the ECOG scale (147/175, 84.1%) and Karnofsky scale (142/175, 81.3%).

**Table 1:** Digital profile of participants (N=175).

Characteristics		n (%)
**Clinician is user of a social network**		
	Yes	174 (99.43)
	No	1 (0.57)
**Operating system of your private mobile**		
	iOS	99 (56.57)
	Android	76 (43.43)
**Apps already downloaded on your mobile**		
	Number of apps (n=123)	24 (19.5)
	Clinical use only (n=144)	5 (20)
**Use of mobile services** **(News/Press/Online journals)**		
	Social networks (eg, Facebook, LinkedIn, Twitter)	146 (83.43)
	Instant messaging (eg, WhatsApp, Snapchat)	119 (68)
	Email	154 (88)
	Online banking	162 (92.57)
	Information of interest	122 (69.71)
	Never used	0 (0)

Participants responded to the question about utility of each corresponding app tool, indicating that the utility of all app tools was considered as highly important (5-7 scored) on a Likert scale (71%-87% of panelists). Whenever these tools were considered of little use, this fact was highly attributable (80%-90% of panelists) to a lack of need during the patient’s examination, although the tools might be used in further visits.

### Acceptability and Usefulness of the App

The level of acceptability for App INES·DIO was tested among the sample. By the end of testing, all participants (N=175) had gained some experience with the system and the mobile app features. Most clinicians (157/175, 89.7%) concluded that the mobile app is well designed and easy to use, and 94.9% (166/175) of participants would likely/most likely recommend the use of App INES·DIO.

A report including all the information collected by physicians from each patient was provided by the app. This report was always/almost always indicated as being worth including in the patient’s clinical records by 68% (119/175) of panelists (Table 2). The app was used as often as two or three times a week by 41.7% (73/175) of clinicians, and it would even be worth using it in another patient’s profile (ie, not exclusively in cancer) to assess the diagnosis and control of pain (Table 2).

**Figure 4:**
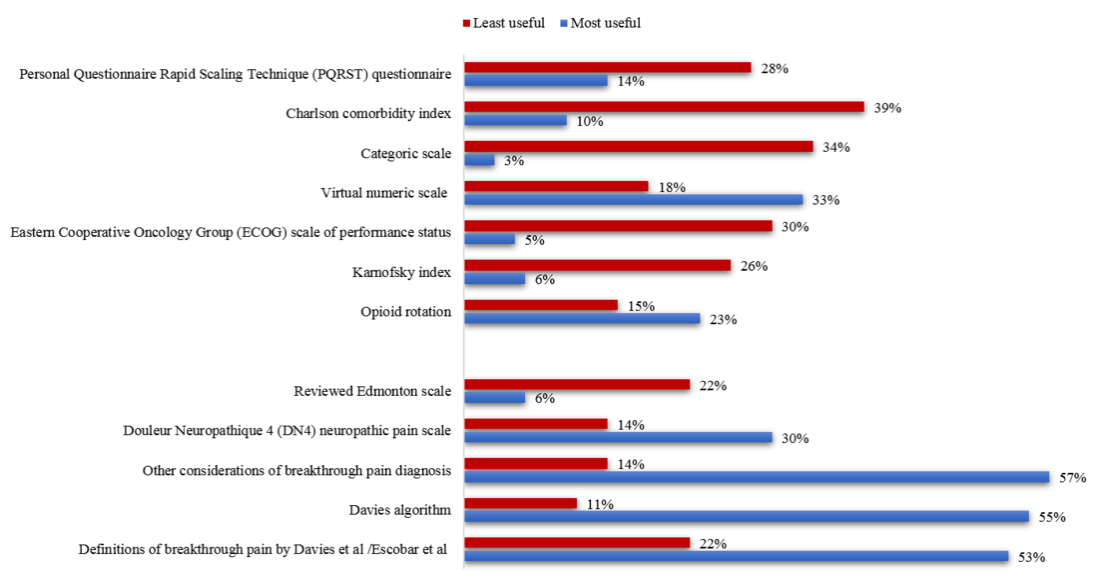
App INES·DIO tools rated for clinical usefulness by professionals.

Clinicians were questioned about the usefulness of each app tool for the diagnosis and monitoring of patients with BTcP. Both most and least useful app features are shown in Figure 4. Davies and Escobar definitions of BTcP (93/175, 53.1%), the use of Davies diagnostic algorithm (96/175, 54.9%) and other considerations for a better diagnosis of BTcP (eg, the number of flares per day, their duration and intensity as well as the control of baseline pain) (100/175, 57.1%), were understood as the most useful tools of App INES·DIO. Conversely, the least useful tools valued by professionals were the ECOG Scale (52/175, 29.7%), the Categorical Scale (59/175, 33.7%), and the Charlson Comorbidity Index (68/175, 38.9%).

**Figure 5:**
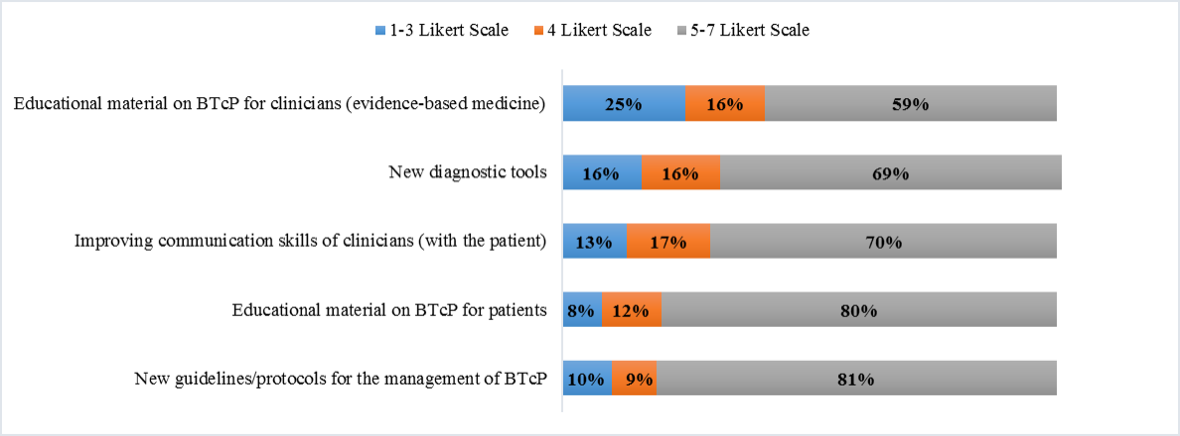
Content proposed by participants for a future version of app. BTcP: breakthrough cancer pain.

The feedback about future content (five different proposals) to be included in the app given by participants who used the App INES·DIO takes the format of a statement based on a fully anchored 3-point Likert-type response, with options being “Disagree” (score 1-3), “Undecided” (score 4), and “Agree” (score 5-7). The sample strongly suggested the implementation of new educational material for patients about the pathology and treatment of breakthrough pain (Figure 5). They strongly agreed on the importance (mostly scores between 5-7) of adding new clinical guidelines/protocols for the management of BTcP, improving their communication skills with the patient, and introducing an evidence-based medicine video platform to gather new educational material on BTcP.

**Table 2:** Usability testing of the app (N=175 clinicians who used each resource).

Type of testing				n (%)
**Usability testing of the BTcP^a^** **diagnostic workflow**				
	Definitions of BTcP by Davies et al [6]/Escobar et al [16]		146 (83.4)	
	Davies algorithm		141 (80.7)	
	Other considerations of BTcP diagnosis		46 (26.4)	
	DN4 neuropathic scale^b^		122 (69.5)	
	Reviewed Edmonton scale^b^		111 (63.4)	
**Usability testing of the app’s pain tools**				
	Opioid rotation		92 (52.8)	
	Karnofsky scale^c^		142 (81.3)	
	ECOG^d^ scale of performance status^c^		147 (84.1)	
	Visual Numeric Scale^e^		163 (93.3)	
	Categorical Scale^e^		124 (70.8)	
	Charlson Comorbidity Index		97 (55.4)	
	PQRST^f^ questionnaire		111 (63.7)	
**Usability testing of App INES·DIO**				
	**Would you include the app report with the clinical history of the patient?**			
		Always	65 (37.1)	
		Almost always	54 (30.9)	
		Occasionally	52 (29.7)	
		Never	4 (2.3)	
	**Would you use App INES·DIO in a different patient profile?**			
		Most likely	70 (40.0)	
		Likely	52 (29.7)	
		Least likely	49 (28.0)	
		Unlikely	4 (2.3)	
	**How many days have you used App INES·DIO on average?**			
		Everyday	31 (17.7)	
		4-6 times per week	38 (21.7)	
		2-3 times per week	74 (42.3)	
		Once per week	32 (18.3)	

^a^BTcP: breakthrough cancer pain.

^b^Tools to assess baseline pain.

^c^Functional scales.

^d^ECOG: Eastern Cooperative Oncology Group.

^e^Pain scales.

^f^PQRST: (P) provocative and palliative factors; (Q) qualitative description of pain; (R) region and radiation of pain; (S) severity or intensity of pain after being scored by means of VNS and CS; (T) timing or pain changes over time.

## Discussion

### Principal Findings

Mobile devices are continuously present in people’s everyday lives [19], and many individuals are firmly tied to their mobile phones, which are typically customized to their specific needs [20,21]. Evolving technical capabilities of mobile devices enable delivery of various services independent of users’ time and place, and their dynamic adaptation to current context of use and users’ personal preferences [22]. These features make mobile devices well-suited platforms for apps that allow easier monitoring and managing of pre-existing health conditions, the delivery of more efficient individually tailored care at the point-of-need, and promotion of a better collaborative work between patients and health care providers [23,24].

To our knowledge, this was the first study to report on the development, usability, usefulness, and acceptability testing of a mobile app to be used as an adjunct to BTcP intervention. Given the popularity of mobile apps within our sample (Table 2) and the difficulties related to management of BTcP, we anticipated that a mobile app would be a useful tool to assist in the diagnosis and monitoring of patients with BTcP and the results of this study support this. This study included the participation of a group of medical experts in the iterative development process. They were selected to achieve a fair distribution across the four professional profiles involved in the management of BTcP: medical oncology, radiation oncology, palliative care, and pain.

Most of the sample recruited was aware of the consensus and controversies driven by the original Delphi study [8] that had set the groundwork for the development of App INES·DIO and the subsequent usability testing described in this study. Moreover, conclusions reported by the Delphi study were perceived to have a positive impact on clinical daily practice when attending BTcP patients.

Ultimately, there has been a rapid proliferation of mHealth apps, and for pain in particular. As of 2015, around 280 apps were commercially available to monitor and track pain [23,24]. In our study, App INES·DIO was tested by 175 professionals in 700 cancer patients with a mean historical diagnosis of BTcP of 2 years. This result is particularly important, mainly because only 8.2% of these reported apps included a health care professional in their development, not a single app provided a theoretical rationale, and only 1 app has undergone scientific evaluation [24].

In the literature, the treatment of BTcP involves strategies such as the treatment of cancer disease, modification of the baseline analgesic treatment, nonpharmacological interventions, and an appropriate rescue medication [25]. In line with this, our app was mostly used to help physicians during the examination of patients, but also the diagnosis, course, and treatment of BTcP flares.

Some authors support the fact that pain history should include key elements that characterize the salient clinical features of breakthrough pain, in addition to standard approaches to cancer pain history [26]. Clinicians were requested to create a new profile with each patient (4 per clinician), going stepwise through the different validated tools incorporated into App INES·DIO to complement the patient’s pain history. Testing the usability of these tools revealed that the BTcP definitions of Davies [6] and Escobar et al [16] and the Davies algorithm were the most used. One of the difficulties attributed to assessing the prevalence of BTcP in the cancer population lies in the variety of definitions that exist and are used for BTcP.

Furthermore, both pain-rating and functional assessment scales were highly used by clinicians, with a peak of 93.3% for the VNS followed by the use of ECOG scale (84.1%) and the Karnofsky scale (81.3%). These results are in line with other research on the use of these scales to test the control of baseline pain [26]. To consider baseline pain as adequately controlled, some authors assume that the average intensity of pain must be <4 on a categorical scale or somewhere on the VNS from 0-10 (0=no pain at all, 10=the worst pain ever possible). Numerical rating scales have shown high correlations with other pain-assessment tools in several studies [27,28], and the feasibility of its use and good compliance have also been proven [29].

During our study, the sample stated the usefulness of each app tool. Interestingly, the tool “Other considerations for the diagnosis of BTcP” was considered as the most useful, even above the use of the Davies diagnosis algorithm. In other words, the diagnosis of BTcP was interpreted by the sample to comprise those features that complete the information related to the definition of an episode: number, duration, and intensity of flares per day and the management of baseline cancer.

Comparing our study against others [23,24], it is clear that the acceptability and usefulness testing done by physicians is critical for the optimal design and development of mobile apps used in clinical cohorts. With regard to user satisfaction, 90% of clinicians reported that they liked using this pain app and found it user friendly and well designed, while 95% reported that they would likely/most likely recommend it to other colleagues, even for use with other patient profiles. The generation of a report that physicians could attach to the clinical record of each patient was considered of great value.

Participants gave feedback about five different types of content to be included in future versions of App INES·DIO for a better interpretation of BTcP. Interestingly, the feedback revealed the need for future implementation of new educational material about the pathology and treatment of breakthrough pain. The sample agreed on adding some new educational tools to the app, such as consensus documents and clinical guidelines for the management of BTcP, improving their communication skills with the patient, and evidenced-based medicine platforms. Refreshing the knowledge and communication skills of health care providers may yield more favorable patient outcomes.

### Limitations

The most significant limitation of this study was the use of a one-group design to pilot App INES·DIO. This design precluded assessment of the feasibility of randomization procedures, as well as recruitment, attrition, outcome measure completion, and acceptability in a control arm. However, although we can learn a lot about the usability of a mobile app in a controlled setting, it is important to test it in real-world situations, which are highly variable [30,31].

### Future Work

Previously, we noted that there was limited related research on how mobile devices could be used in the context of health care information systems for cancer patients. Further work is needed to identify the primary factors and design issues influencing acceptability and usefulness of different system features of mHealth care information services. In our future research, we are planning to continue work on the development of a new version of App INES·DIO and investigate how this app should be designed and adjusted to best fit clinicians’ needs in the care of BTcP patients. Some of the potential new app features were already identified throughout this survey study, and these will be considered for next version of the app, as well as the need for further exploration of how we can add rich media to this BTcP mobile app.

### Conclusions

In summary, these results suggest that App INES·DIO could soon be used as a tool to help physicians make decisions around BTcP management. Indeed, this app can be a reference medical device to assess the diagnosis and monitoring of BTcP. Clinical use of diagnostic tools going beyond the Davies algorithm should be outlined in any patient with a history of cancer pain. The value of the app will be enhanced with the inclusion of new educational material on BTcP not only for medical professionals but also for patients.

## Abbreviations

App INES·DIO: (abbreviation in Spanish) Instrument for the Assessment and Monitoring the Breakthrough Cancer Pain
BTcP: breakthrough cancer pain
CS: Categorical Scale
ECOG: Eastern Cooperative Oncology Group
PQRST: (P) provocative and palliative factors; (Q) qualitative description of pain; (R) region and radiation of pain; (S) severity or intensity of pain after being scored by means of VNS and CS; (T) timing or pain changes over time
VAS: Visual Analogue Scale
VNS: Visual Numeric Scale
